# Neonatal modification of endocrine functions and mammary carcinogenesis in the rat.

**DOI:** 10.1038/bjc.1976.122

**Published:** 1976-07

**Authors:** S. Christakos, D. Sinha, T. L. Dao

## Abstract

Effects of neonatal androgenization or neonatal ovariectomy in female rats on endocrine functions and mammary tumourigenesis are examined. Pituitary gonadotrophin contents (both LH and FSH) are significantly lower in neonatally androgenized rats (TT) and significantly increased in neonatally ovariectomized rats (NO) when compared with controls of the same age. Plasma and pituitary prolactin levels are higher in TT rats than in the control rats of the same age, but the difference is not significant. Mammary tumours developing in TT rats after DMBA treatment are predominantly fibroadenomata, and lactogenesis in TT rats occurs almost entirely in those receiving DMBA treatment. Neonatal ovariectomy in female rats protects against subsequent induction of mammary cnacer by DMBA. The relationship between neonatal modification of endocrine functions and mammary tumourigenesis is discussed.


Br. J. Cancer (1976) 34, 58

### NEONATAL MODIFICATION OF ENDOCRINE FUNCTIONS AND MAMMARY CARCINOGENESIS IN THE RAT

S. CHRISTAKOS*, D. SINHA AND T. L. DAO†

*From the Department of Breast Surgery and Breast Cancer Research Unit, Roswell Park Memorial Institute, Buffalo, New York 14263*

Received 6 February 1976 Accepted 22 March 1976

**Summary.—Effects of neonatal androgenization or neonatal ovariectomy in female rats on endocrine functions and mammary tumourigenesis are examined. Pituitary gonadotrophin contents (both LH and FSH) are significantly lower in neonatally androgenized rats (TT) and significantly increased in neonatally ovariectomized rats (NO) when compared with controls of the same age. Plasma and pituitary prolactin levels are higher in TT rats than in the control rats of the same age, but the difference is not significant. Mammary tumours developing in TT rats after DMBA treatment are predominantly fibroadenomata, and lactogenesis in TT rats occurs almost entirely in those receiving DMBA treatment. Neonatal ovariectomy in female rats protects against subsequent induction of mammary cancer by DMBA. The relationship between neonatal modification of endocrine functions and mammary tumourigenesis is discussed.**

A SINGLE injection of testosterone given to female rats during the first 10 days of life induces permanent morphological and functional changes in the gonadotrophin–ovarian axis. Endocrine abnormalities such as small polyfollicular ovaries lacking corpora lutea, low pituitary luteinizing hormone content, low plasma oestrogen level and constant vaginal cornification, have been regularly observed in these neonatally testosterone-treated (TT) rats (Barraclough, 1967; Gorski and Barraclough, 1962; Mallampati and Johnson, 1973).

The effect of these hormonal alterations on mammary tumourigenesis, however, has not been examined in depth, even though ovarian and pituitary hormones are critically involved in the induction of mammary tumours in the rat (Clemens, Welsh and Meites, 1968; Dao, 1962). In this investigation, the effects of neonatal testosterone treatment and neonatal ovariectomy on pituitary and gonadal function are examined in detail and are correlated with the effect of the carcinogen 7,12-dimethylbenz($\alpha$)-anthracene (DMBA) on the induction of mammary tumours.

### MATERIALS AND METHODS

*Animals.*—A total of 150 Sprague–Dawley female rats (Holtzman Company, Madison, Wisconsin) were used in this study. All newborns used for this investigation were obtained in our own laboratory. The animals were kept under conditions of rhythmic artificial illumination (14 h/day) and were housed in a temperature-controlled room (22 $\pm$ 1 °C). A diet of Rockland pellets and water was provided *ad libitum.*

*Experimental.*—Female rats were neonatally sterilized by either a single s.c. injection of 1·25 mg testosterone propionate dissolved in 0·05 ml olive oil within 24 h of their birth, or by the removal of the ovaries at 5 days of age. At 55 days of age, 32 neonatally testosterone-treated (TT), 10 neonatally ovariectomized (NO), and 30

* Portions of this work are taken from a thesis submitted in fulfilment of the requirements for Ph.D. degree in Physiology at SUNY at Buffalo, Roswell Park Graduate Division, Buffalo, N.Y.

† To whom requests for reprints should be addressed.

untreated control rats were given a single i.v. injection of 1 ml of a lipid emulsion containing 5 mg of DMBA. These animals were examined for tumours once weekly. Tumours were measured in two dimensions with a vernier caliper. At the time of autopsy (90 days after carcinogen administration), tumours were removed, fixed in Bouin's solution, and sections prepared and stained with haematoxylin and eosin. For comparison, 10 TT, 10 NO, and 10 control rats without DMBA treatment were sacrificed at 60 and 150 days of age. Vaginal smears were taken regularly in control animals and, when possible, in NO and TT rats, since most of them have pinpoint vaginal openings. Blood was collected for prolactin assay by cardiac puncture under ether anaesthesia using a heparinized needle and syringe. Control pituitaries were collected when the rats were in oestrus since TT rats have a constant oestrus vaginal smear pattern, due to their anovulatory polyfollicular state (Barraclough, 1967). The anterior pituitaries were individually weighed, and both plasma and anterior pituitaries were frozen immediately at −20°C until the time of prolactin assay. In another set of experiments, pituitary gonadotrophins including FSH and LH in TT and NO rats were compared with that in the control rats. Morphology of the mammary gland and the gonads from these three groups of animals was also studied.

*Prolactin assay.*—Prolactin of individual plasma samples and pituitaries was measured by a radioimmunoassay described previously by Niswender *et al.* (1969). Sheep anti-rabbit globulin was purchased from Grand Island Biological Company (Grand Island, New York). Anti-rat-prolactin reference standard and rat prolactin for iodination were kindly supplied by the National Institute of Arthritis, Metabolic and Digestive Diseases (rat pituitary distribution programme). Data from radioimmunoassay were subjected to analysis of variance.

*Assay of pituitary FSH and LH.*—Pituitary luteinizing hormone (LH) content was determined by the method described by Parlow (1961). This method is based on the ability of LH to deplete ascorbic acid in the copora lutea of immature (21–22-day-old) pseudopregnant rats. The assay is specific since other pituitary trophic hormones, such as follicle stimulating hormone, prolactin and ACTH, have no effect on ascorbic acid content in the corpora lutea.

Pituitary follicle stimulating hormone (FSH) was assayed by the modification (Parlow and Reichert, 1963) of the method originally described by Steelman and Pohley (1953). This method is based on the increase in ovarian weights after injection of pituitary extracts in 21–22-day-old female rats. The standard curve is constructed by the changes in ovarian weights in response to injections of a mixture of standard FSH (NIH-FSH-S8) at different dose levels and 50 iu of HCG. HCG is used to augment the increase in ovarian weights induced by FSH.

### RESULTS

### *Morphological studies of the mammary glands, gonads, and pituitaries in TT, NO and control rats*

There is no difference in the morphology of mammary glands in TT and control rats. In NO rats the mammary glands are atrophic. The weights of the ovaries, uteri, adrenal glands and pituitaries in TT and NO rats at 60 and 150 days and their respective controls are summarized in Table I. The ovarian

TABLE I.—*Endocrine Organs in Control, Neonatally Androgenized (TT) and Neonatally Ovariectomized (NO) Rats*

| Age (days) | Group | Mean organ weight ± standard error (mg/100 g) Ovary | Uterine horn | Adrenal | Pituitary |
|---|---|---|---|---|---|
| 60 | Control | 30·90±1·19 | 147·60±5·92 | 29·9±0·79 | 8·7±0·42 |
| 60 | TT | 12·10±0·59 | 102·90±4·29 | 25·1±0·91 | 10·2±0·86 |
| 60 | NO | — | 21·1±3·19 | 23·0±1·49 | 9·1±0·62 |
| 150 | Control | 36·30±1·23 | 232·3±7·13 | 35·9±0·87 | 11·9±1·19 |
| 150 | TT | 15·50±1·33 | 149·3±5·87 | 35·2±1·17 | 14·8±0·89 |
| 150 | NO | — | 23·9±1·34 | 32·9±0·69 | 14·1±0·42 |

and uterine weights in TT rats at both 60 and 150 days are significantly lower than their respective controls ($P < 0 \cdot 001$). The adrenal weights show no change as a result of neonatal treatment with testosterone or removal of the ovaries. The pituitaries of TT and NO rats are larger than their controls at 150 days, but these differences are not significant.

Histological examination reveals that ovaries of TT rats contain follicles but no corpus luteum. Occasionally, atretic follicles are visible in the sections. Uterine horns are smaller in the TT rats as compared with those of normal females of the same age. Histological examination reveals that the uterine epithelial layer in TT rats is composed of tall columnar epithelial cells. In addition, islands of squamous metaplasia are frequently seen. The endometrial stroma is compact and has few glands. However, the myometrium of the uterine horns is similar in appearance to that of normal females. NO rats have atrophic uteri. Both endometrium and myometrium are thin and practically no glands have ever been observed in these sections. The pituitaries from TT rats are morphologically similar to those from the controls. Those from NO rats, however, have markedly hypertrophied basophils, a typical castration effect.

*Pituitary gonadotrophin content in NO, TT and control rats*

The results are summarized in Table II. Pituitary luteinizing hormone concentration in TT rats is about 50% that of normal females. This is true both at 60 ($P < 0 \cdot 01$) and 150 ($P < 0 \cdot 001$) days. LH concentration in the pituitary of NO rats, however, is increased 7- and 5-fold over normal females at 60 and 150 days of age, respectively. FSH content is lower in the pituitaries of TT rats than in the control rats, but the difference is significant only at 150 days. There is a significant increase of FSH in the pituitaries of NO rats.

*Pituitary and plasma prolactin concentrations in controls, TT and NO rats*

Results shown in Table II demonstrate that the pituitary prolactin levels in TT rats are slightly lower than the control oestrus females at both 60 and 150 days. These differences, however, are not statistically significant. The pituitary prolactin in the NO rats, however, is significantly lower than in the control rats ($P < 0 \cdot 001$ at 60 days and $P < 0 \cdot 005$ at 150 days). Treatment with DMBA does not induce any significant change in the level of pituitary prolactin in either the control or TT rats at 150 days. The figure shows that plasma prolactin concentration is higher in TT rats than in the controls at both 60 and 150 days. This apparent increase in plasma prolactin concentration, however, is not statistically significant. The NO rats, on the other hand, have plasma prolactin levels that are 25% of the control value at 60 days ($P < 0 \cdot 001$) and 20% of the control value at 150 days ($P < 0 \cdot 005$). The plasma prolactin concentrations in rats given

TABLE II.—*Pituitary Gonadotrophin and Prolactin Content in NO, TT, and Control Rats*

| Age (days) | Group | LH (μg/mg pit.) | FSH (μg/mg pit.) | Prolactin (μg/mg pit.) |
|---|---|---|---|---|
| 60 | Control | 1·87±0·26 | 4·9±0·31 | 2·3±0·41 |
| 60 | TT | 0·96±0·14† | 3·7±0·46 | 1·9±0·90 |
| 60 | NO‡ | 13·11±2·31 | 26·0±4·10 | 0·3±0·01* |
| 150 | Control | 2·17±0·04 | 4·1±0·26 | 5·95±0·92 |
| 150 | TT | 1·03±0·008* | 2·4±0·30† | 5·60±0·55 |
| 150 | NO‡ | 11·82±1·16 | 25·2±5·10 | 0·32±0·07† |

* $P < 0 \cdot 001$.
† $P < 0 \cdot 01$.
‡ Both LH and FSH are significantly higher than control or TT rats of the same age group. $< 0 \cdot 001$.

FIG.—Plasma prolactin levels in Control, TT and NO rats at 60 and 150 days of age, and DMBA treated rats (Control and TT) at 150 days.

DMBA are not significantly different from those of untreated controls and the untreated TT rats at 150 days. Also, there does not seem to be any correlation between tumour size and plasma prolactin. It should be noted that both plasma and pituitary prolactin levels rise with the increase in the ages of rats in the control as well as TT groups. This observation is in agreement with the earlier report by Voogt, Chen and Meites (1970).

*Tumourigenesis of the mammary gland in control, TT and NO rats*

The incidence of tumour induction after DMBA treatment is less in TT rats than in controls. Results in Table III show that 20 out of 32 TT rats developed tumours after DMBA treatment as compared to 28 of 30 control rats. However, the mean number of tumours per rat was the same and the growth rate of tumours in the TT rats did not differ markedly from that of the control rats. DMBA failed to induce any tumours in the NO rats.

The latent period for tumour appearance in TT rats is longer than that in the controls. An average of 15 weeks for the first appearance of a tumour was observed after DMBA treatment in TT rats as compared with 11·3 weeks in controls. This difference is statistically significant ($P < 0{\cdot}001$).

The histology of DMBA-induced mammary tumours in the control and TT rats was examined and compared. There was a greater incidence of fibroadenomata in TT rats (65%) as opposed to the greater incidence of adenocarcinomata in the control rats (100%) (Table III). The finding that fibroadenomata were the predominant tumours in TT rats agrees with the work of Shellabarger and Soo (1973).

One interesting observation in this study is the induction of lactogenesis in the mammary gland of TT rats (81%) receiving DMBA treatment. This finding was not made in the normal controls given DMBA or in TT rats not given DMBA.

TABLE III.—*Incidence and Histology of Mammary Tumours Induced by DMBA in the Control and Neonatally Androgenized Rats*

| Groups | No. of rats | No. of rats with tumours | Tumour histology: Adeno-carcinoma | Fibro-adenoma |
|---|---|---|---|---|
| Control | 30 | 28 | 28 | 0 |
| TT | 32 | 20 | 7 | 13 |
| NO | 10 | 0 | 0 | 0 |

DISCUSSION

The effect of neonatal androgenization on pituitary gonadotrophin and prolactin in female rats has been studied by several investigators. Barraclough (1967) observed a significant reduction in pituitary LH but not in pituitary FSH in TT rats. Our findings, however, show that both LH and FSH in the pituitary of TT rats are significantly lower than the control levels. These results are in agreement with data reported by others (Johnson and Witschi, 1963; Shapiro, 1965). The difference between Barraclough's data and those reported in this paper and by other investigators may be attributable to the difference in methods used for assay of FSH. Barraclough used a bioassay method which may be less sensitive than the method of Steelman and Pohley used by other investigators.

It has been suggested that reduction in LH concentration in the pituitary is the result of a permanent effect in the hypothalamus induced by a neonatal injection of testosterone. Thus, Gorski and Barraclough (1962) were unable to induce ovulation in TT rats by electrical stimulation of the hypothalamus. However, marked decrease in pituitary LH content has also been observed in rats following destruction of the anterior hypothalamus (Cheng and Johnson, 1974).

Both TT rats, and rats whose anterior hypothalamus has been destroyed, have persistant oestrus, non-ovulating polyfollicular ovaries and, thus, constant oestrogen secretion. It is possible that oestrogen feedback on the hypothalamus prevents storage of LH in the pituitary. In contrast, in NO rats, both LH and FSH in the pituitary increase several fold due mainly to a lack of hormonal feedback causing release of these gonadotrophins.

The results obtained in this investigation using radioimmunoassay show that there is an apparent increase in serum prolactin in TT rats at 60 days, above the level in control rats of the same age. The difference, however, is not statistically significant. This finding is in agreement with the earlier report by Mallampati and Johnson (1973). In the present study, the pituitary prolactin contents in TT rats at both 60 and 150 days are lower than those in the control rats of the same age, a finding similar to that reported by Kurcz *et al.* (1967). Altogether, these results suggest that androgenized rats have normal prolactin synthesis and release. This interpretation is supported by an observation of the normal development of mammary glands in the TT rats as determined by histological examination, and by the normal synthesis of casein by the mammary gland of the TT rats (unpublished data). In the NO rat, the absence of a feedback of gonadal oestrogen on the hypothalamus and pituitary causes a markedly reduced prolactin synthesis and release.

The critical role of oestrogen in initiating the induction of mammary tumours by a chemical carcinogen has been reported earlier (Dao, 1962). In this investigation, it is shown that neonatal ovariectomy completely prevents mammary carcinogenesis in these rats, when adult, and neonatal testosterone treatment causes a reduction of mammary tumour in adult. Our results are in agreement with reports by other investigators (Shellabarger and Soo, 1973; Dao, 1966; Kovac, 1965). In addition, we also observe a striking difference in the histopathology of the tumours which develop in the TT rats as compared to those in the control rats. The finding that fibroadenomata are the predominant tumours in TT rats cannot be readily explained. It seems that cyclic ovarian function is essential for induction of mammary adenocarcinoma, since this cycling is absent after neonatal testosterone treatment.

Lactogenesis in testosterone-sterilized rats was first reported by Dao (1966) and later confirmed by Stern, Mickey and Osvald (1967) and by Shellabarger and Soo (1973). Dao and Greiner (1961) also observed lactogenesis in castrated male rats bearing ovarian grafts given 3-methylcholanthrene. In this present investigation, we find that lactogenesis in the mammary gland develops in TT rats only after DMBA treatment, and not in those not given DMBA. Since TT or castrated male rats with ovarian grafts have noncyclic ovaries, one would expect a constant prolactin secretion from a noncyclic pituitary. DMBA may initiate lactation by an effect on the hypothalamus (Dao and Sinha, 1975), or it may exert a direct effect on the mammary gland cells, rendering them more sensitive to lactogenesis by circulating prolactin. Work is now being carried out in our laboratory to elucidate these possibilities.

This study was supported by a grant (CA 14812-1) from the National Cancer Institute.

### REFERENCES

Barraclough, C. (1967) Modifications in Reproductive Functions after Exposure to Hormones during the Prenatal and Early Postnatal Period. In *Neuroendocrinology*, **2**. Ed. L. Martini and W. Ganong. New York: Academic Press.

Cheng, H. C. & Johnson, D. C. (1974) Serum Estrogen Gonadotropins in Developing Androgenized and Normal Female Rats. *Neuroendocrinology*, **13**, 357.

Clemens, J., Welsh, C. & Meites, J. (1968) Effects of Hypothalamic Lesions on Incidence and Growth of Mammary Tumors in Carcinogen Treated Rats. *Proc. Soc. exp. Biol. Med.*, **127**, 969.

Dao, T. L. (1962) The Role of Ovarian Hormones in Initiating the Induction of Mammary Cancer in Rats by Polynuclear Hydrocarbons. *Cancer Res.*, **22**, 973.

Dao, T. L. (1966) Mammary Tumorigenesis in Female Rats Receiving Androgen Neonatally. *Proc. Am. Assoc. Cancer Res.*, **7**, 16.

Dao, T. L. & Greiner, M. (1961) Mammary Carcinogenesis by 3-Methylcholanthrene. Induction of Mammary Carcinoma and Milk Secretion in Male Rats Bearing Ovarian Grafts. *J. natn. Cancer Inst.*, **27**, 333.

Dao, T. L. & Sinha, D. (1975) Effect of Carcinogen on Pituitary Prolactin Synthesis and Release. *Proc. Am. Assoc. Cancer Res.*, **16**, 28.

Gorski, R. A. & Barraclough, C. (1962) Adenohypophyseal LH Content in Normal, Androgen-sterilized and Progesterone-primed Sterile Rats. *Acta Endocrin.*, **39**, 13.

Johnson, D. S. & Witschi, E. (1963) Hypophyseal Gonadotropins Following Gonadectomy in Male and Female Androgenized Rats. *Acta endocrin.*, **44**, 119.

Kovac, K. (1965) Effect of Androgenic Action on the Development of Mammary Tumors in Rats Induced by Oral Administration of 9,10-Dimethyl-1,2-benzanthracene. *Br. J. Cancer*, **19**, 531.

Kurcz, M., Kovacs, K., Tiboldi, T. & Orasz, A. (1967) Effect of Androgenization on Adenohypophyseal Prolactin Content in Rats. *Acta endocrin.*, **54**, 663.

Mallampati, R. & Johnson, D. C. (1973) Serum and Pituitary Prolactin, LH and FSH in Androgenized Female and Normal Male Rats Treated with Various Doses of Estradiol Benzoate. *Neuroendocrinology*, **11**, 46.

Niswender, D., Chen, C. L., Midgley, A., Meites, J. & Ellis, S. (1969) Radioimmunoassay for Rat Prolactin. *Proc. Soc. exp. Biol. Med.* **130**, 793.

Parlow, A. F. (1961) Bioassay of Pituitary Luteinizing Hormone by Depletion of Ovarian Ascorbic Acid. In *Human Pituitary Gonadotropins*. Ed. A. Albert. Springfield, Illinois: C. Thomas.

Parlow, A. F. & Reichart, L. (1963) Influence of Follicle Stimulating Hormone on the Prostate Assay of Luteinizing Hormone. *Endocrinology*, **73**, 377.

Shapiro, S. (1965) Androgen Treatment in Early Infancy. Effect upon Adult Adrenal Cortical Response to Stress and Adrenal and Ovarian Compensatory Hypertrophy. *Endocrinology*, **77**, 505.

Shellabarger, C. & Soo, V. A. (1973) Effects of Neonatally Administered Sex Steroids on 7,12-Dimethylbenz(a)anthracene Induced Mammary Neoplasia in Rats. *Cancer Res.*, **33**, 1567.

Stern, E., Mickey, M. & Osvald, L. (1967) Lactogenesis in the Androgen Sterile Rat; Augmentation Following DMBA. *Nature, Lond.*, **216**, 185.

Steelman, S. & Pohley, F. (1953) Assay of the Follicle Stimulating Hormone Based on the Augmentation with Human Chorionic Gonadotropins. *Endocrinology*, **53**, 604.

Voogt, J. L., Chen, C. L. & Meites, J. (1970) Serum and Pituitary Prolactin Levels Before, During and After Puberty in Female Rats. *Am. J. Physiol.*, **218**, 396.


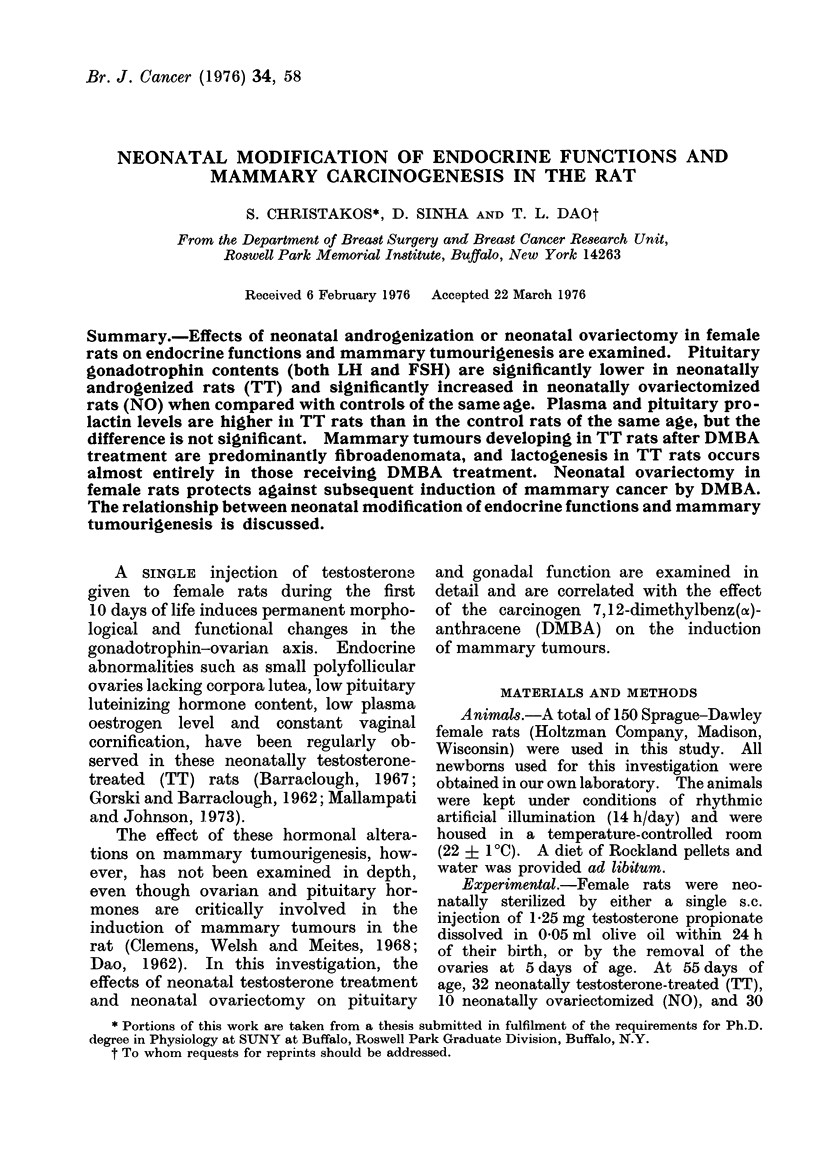

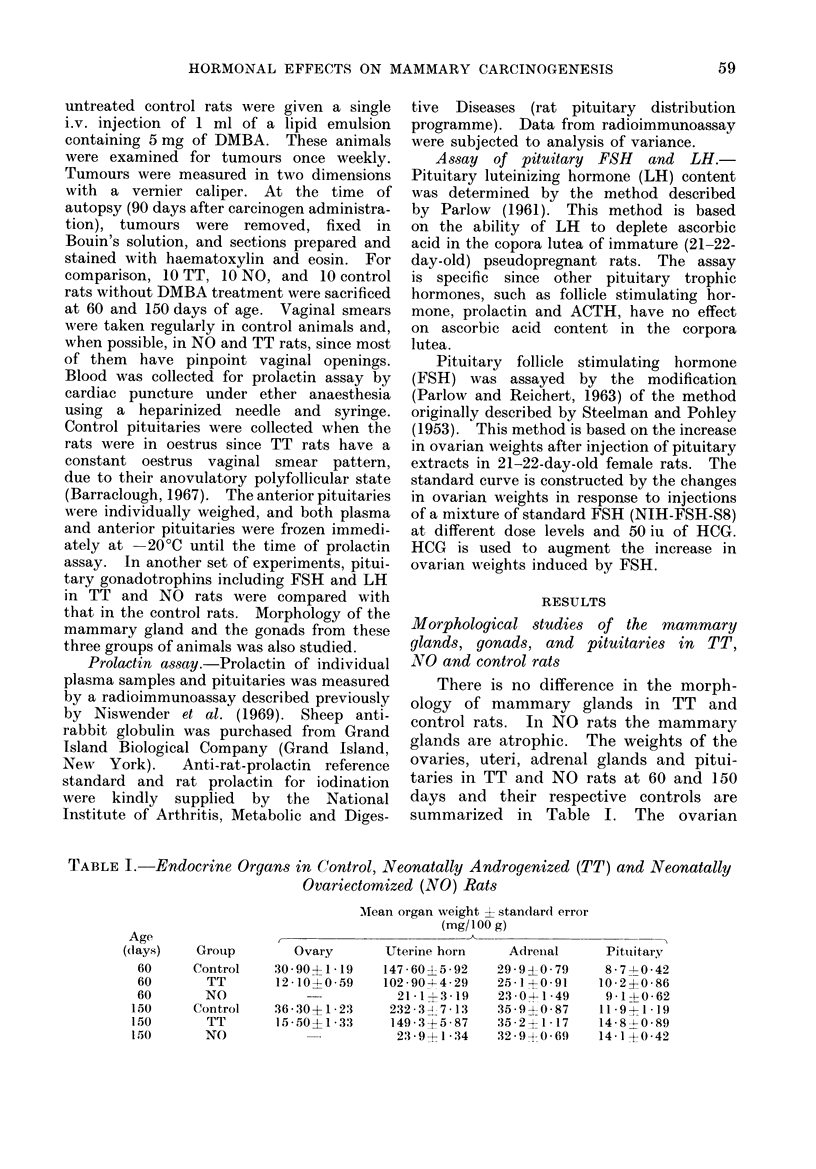

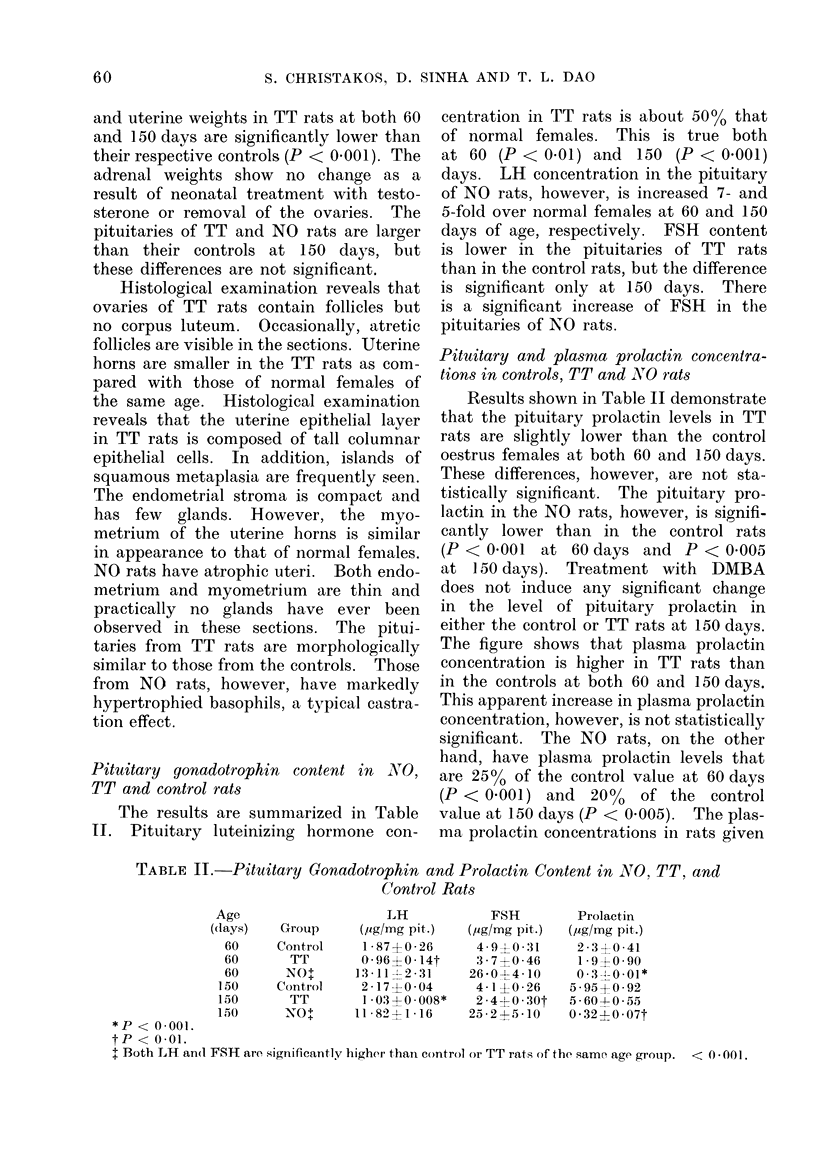

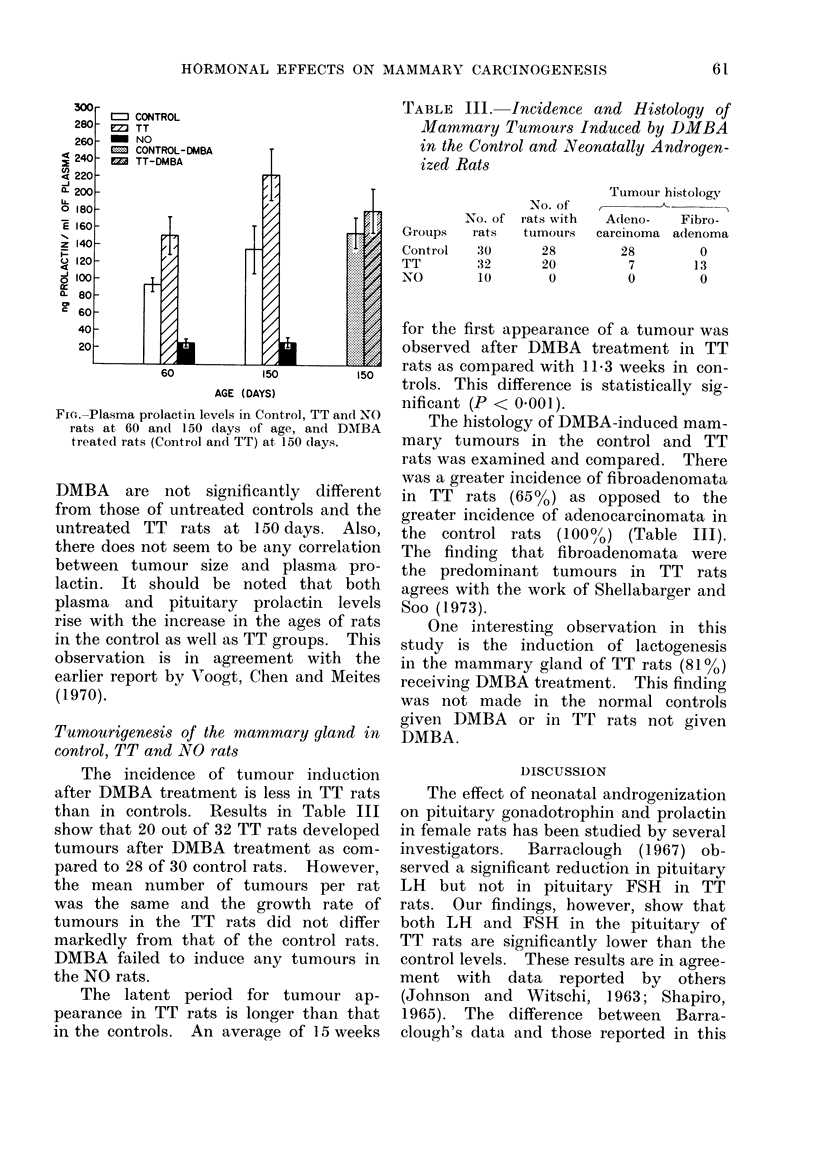

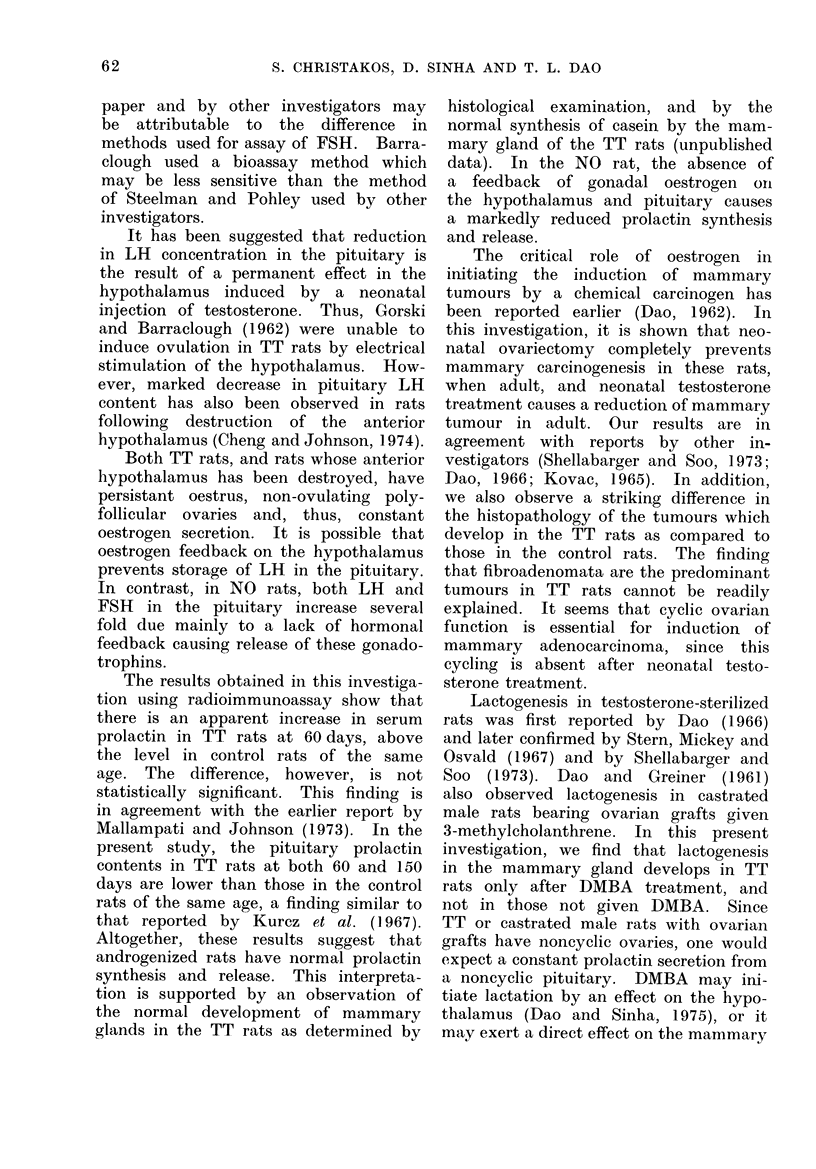

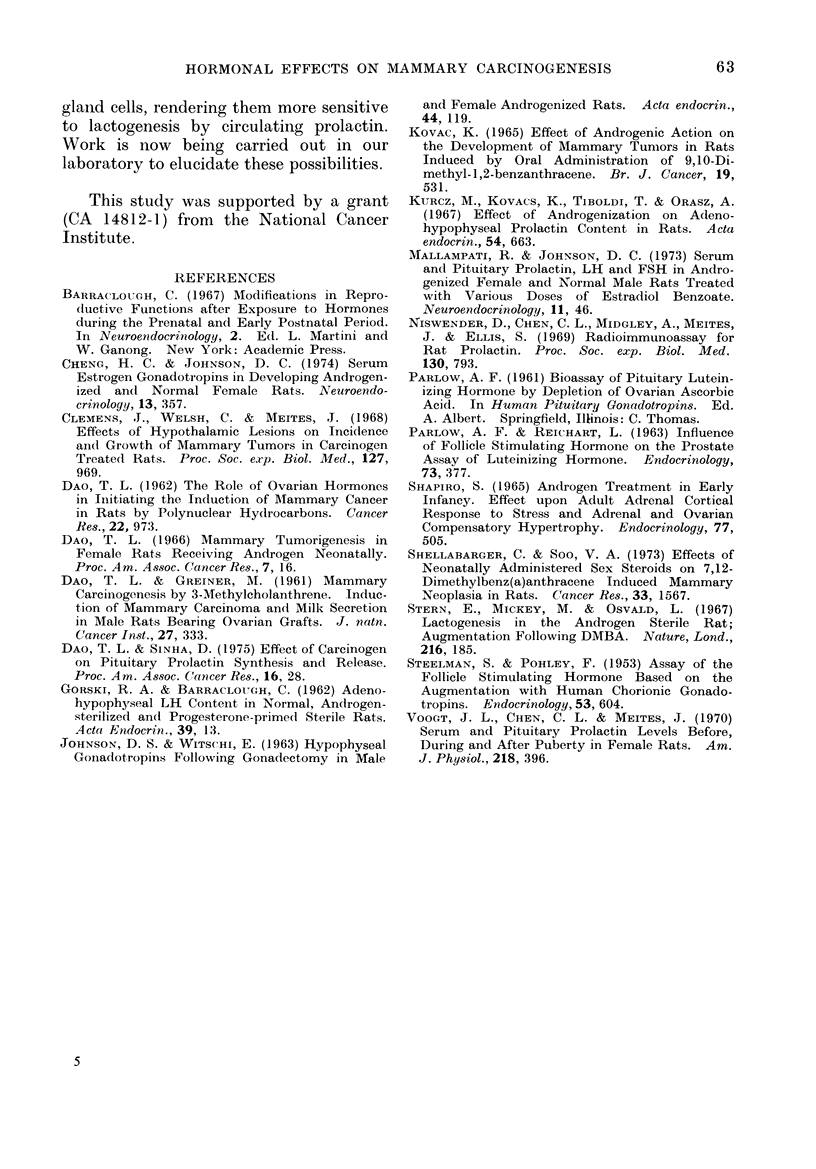


## References

[OCR_00646] Cheng H. C., Johnson D. C. (1974). Serum estrogens and gonadotropins in developing androgenized and normal female rats.. Neuroendocrinology.

[OCR_00652] Clemens J. A., Welsch C. W., Meites J. (1968). Effects of hypothalamic lesions on incidence and growth of mammary tumors in carcinogen-treated rats.. Proc Soc Exp Biol Med.

[OCR_00670] DAO T. L., GREINER M. J. (1961). Mammary carcinogenesis by 3-methylcholanthrene. III. Induction of mammary carcinoma and milk secretion in male rats bearing ovarian grafts.. J Natl Cancer Inst.

[OCR_00659] DAO T. L. (1962). The role of ovarian hormones in initiating the induction of mammary cancer in rats by polynuclear hydrocarbons.. Cancer Res.

[OCR_00682] GORSKI R. A., BARRACLOUGH C. A. (1962). Adenohypophyseal LH content in normal androgen-sterilized and progesteroneprimed sterile female rats.. Acta Endocrinol (Copenh).

[OCR_00688] JOHNSON D. C., WITSCHI E. (1963). HYPOPHYSEAL GONADOTROPHINS FOLLOWING GONADECTOMY IN MALE AND FEMALE ANDROGENIZED RATS.. Acta Endocrinol (Copenh).

[OCR_00695] Kovács K. (1965). Effect of androgenisation on the development of mammary tumours in rats induced by the oral administration of 9,10-dimethyl-1,2-benzanthracene.. Br J Cancer.

[OCR_00702] Kurcz M., Kovács K., Tiboldi T., Orosz A. (1967). Effect of androgenisation on adenohypophysial prolactin content in rats.. Acta Endocrinol (Copenh).

[OCR_00708] Mallampati R. S., Johnson D. C. (1973). Serum and pituitary prolactin, LH, and FSH in androgenized female and normal male rats treated with various doses of estradiol benzoate.. Neuroendocrinology.

[OCR_00715] Niswender G. D., Chen C. L., Midgley A. R., Meites J., Ellis S. (1969). Radioimmunoassay for rat prolactin.. Proc Soc Exp Biol Med.

[OCR_00727] PARLOW A. F., REICHERT L. E. (1963). INFLUENCE OF FOLLICLE-STIMULATING HORMONE ON THE PROSTATE ASSAY OF LUTEINIZING HORMONE (LH, ICSH).. Endocrinology.

[OCR_00752] STEELMAN S. L., POHLEY F. M. (1953). Assay of the follicle stimulating hormone based on the augmentation with human chorionic gonadotropin.. Endocrinology.

[OCR_00740] Shellabarger C. J., Soo V. A. (1973). Effects of neonatally administered sex steroids on 7,12-dimethylbenz(a)anthracene-induced mammary neoplasia in rats.. Cancer Res.

[OCR_00746] Stern E., Mickey M. R. (1967). Neural mechanism in induction of dioestrus and tumour in the androgen sterile rat.. Nature.

[OCR_00758] Voogt J. L., Chen C. L., Meites J. (1970). Serum and pituitary prolactin levels before, during, and after puberty in female rats.. Am J Physiol.

